# The Value of Contrast-Enhanced Transesophageal Echocardiography in the Detection of Cardiac Right-to-Left Shunt Related with Cryptogenic Stroke and Migraine

**DOI:** 10.1155/2020/8845652

**Published:** 2020-12-12

**Authors:** Huiqin Zhang, Wenyan Huang, Tingyu Lan, Meng Zhang, Jing Yang, Hongxia Zhang, Lijuan Du

**Affiliations:** Department of Ultrasound, Beijing Tiantan Hospital, Capital Medical University, 119 West Road of South 4th Ring Road, Fengtai District, Beijing, China 100160

## Abstract

**Purpose:**

To analyze the characteristics of right-to-left shunt (RLS) in patients with cryptogenic stroke and migraine by contrast-enhanced transesophageal echocardiography (c-TEE).

**Methods:**

The study population consisted of 330 patients with cryptogenic stroke and 330 patients with migraine who suspected PFO. All of them received c-TEE examination successfully. In terms of c-TEE analyses, RLS could be diagnosed when microbubbles were visualized in the transition from the right atrium to the left atrium. For semiquantitative analysis, a small amount of RLS was grade 1, indicating 1-10 microvesicles per frame could be seen in the left atrium, a moderate amount of RLS was grade 2, indicating 11-30 microvesicles per frame could be seen in the left atrium, and a large amount of RLS was grade 3, indicating more than 30 microvesicles per frame, or the left atrium is filled with microvesicles.

**Results:**

A total of 660 patients were analyzed in the study. PFO-RLS was detected in 348 (348/660, 52.7%) cases by TEE, while in 392 (392/660, 59.3%) cases by c-TEE. Simultaneously, P-RLS was detected in 239 (239/660, 36.2%) cases by c-TEE. Among 330 patients with cryptogenic stroke, PFO-RLS was detected in 198 cases; according to the c-TTE method (198/330, 60.0%), concurrently, 83 participants suffered from PFO-RLS and P-RSL (83/330, 25.1%), including 1 case with PFO and pulmonary arteriovenous fistula. Among 330 patients with migraine, PFO-RLS was detected in 194 cases; according to the c-TTE method (194/330, 58.7%), specifically, 90 participants suffered from PFO-RLS and P-RSL (90/330, 27.2%). There was no statistical significance between the two groups. P-RLS singly was detected in 28 cases with cryptogenic stroke, while in 38 cases with migraine, excluding from pulmonary arteriovenous fistula by CTA examination. In addition, semiquantitative results on c-TTE grading of RLS were compared between the two groups: grade 1 RLS in the migraine group (144/322) was significantly higher than that in the cryptogenic stroke group (71/309) (*P* < 0.05). Grade 3 RLS in the cryptogenic stroke group (113/309) was significantly higher than that in the migraine group (67/322) (*P* < 0.05). For grade 2 RLS, there was no statistical difference between the two groups (*P* = 0.12).

**Conclusions:**

c-TEE can increase the positive rate of PFO diagnosis compared with TEE color Doppler. There is no significant difference in the incidence of PFO-PLS and P-RLS between the cryptogenic stroke group and the migraine group. The grades 2-3 RLS are mainly detected in the cryptogenic stroke group, while grades 1-2 RLS are mostly detected in the migraine group.

## 1. Introduction

In the past, PFO is deemed as a significant phenomenon that is highly correlated with migraine headaches, cryptogenic stroke, and other common mental disorders. Hence, more and more studies have focused on PFO-RLS, in terms of RLS-related diseases [1, 2]. While in recent years, several studies indicated that other types of RLS, other than PFO-RLS, are not uncommon, with the incidence rate between 20 and 41%. Besides, other types of RLS are mainly originated from the lungs, called as pulmonary RLS (P-RLS), but the underlying mechanisms have been not yet very clear [3–5].

Among patients with a right-to-left shunt, some only have clinical manifestations of migraine, while others can cause more severe symptoms of stroke. It is rarely reported whether there are differences in the characteristics of a right-to-left shunt between these two groups. Contrast transesophageal echocardiography (c-TEE) can distinguish the source of right-to-left shunts and perform semiquantitative analysis of shunts, which is the gold standard [6, 7]. Thus, this research is aimed at investigating the characteristics of PFO-RLS and/or P-RLS via c-TEE in patients with cryptogenic stroke and with migraine, thereby to provide references for clinical practice.

## 2. Materials and Methods

### 2.1. Patient Population

660 consecutive patients including 330 suffered from cryptogenic stroke and 330 suffered from migraine from January 2018 to December 2019 in our hospital who suspected PFO were enrolled. All patients were able to tolerate the TEE examination and perform the Valsalva maneuver in this study. The patients or their relatives provided written informed consent to participate in this study prior to the examination. The study protocol was approved by the Ethics Committee of Beijing Tiantan Hospital, Capital Medical University.

### 2.2. Equipment and Operation Methods

Color Doppler ultrasound diagnostic apparatus (model: Philips iE33 or Philips EPIQ7C) with X7-2t transesophageal probe of 1-15 MHz frequency was utilized to make the diagnosis in this research. All patients were instructed to fast for more than 12 hours, and removable dentures were removed before the examination. Before the TEE procedure, all patients have received 2% lidocaine mucilage for oropharynx anesthesia for 10-15 minutes. The probe was rotated within 45°-110° to clearly display the primary septum and secondary septum and to detect whether an opened PFO and RLS could be found in both two-dimensional and color Doppler ultrasonography.

**Figure 1:**
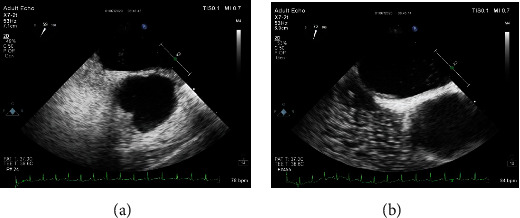
The PFO-RLS and the P-RLS in the same patient with CS. (a) A small amount of RLS from PFO. (b) A moderate amount of RLS from the pulmonary vein.

A TEE bubble examination was conducted to find the presence of a PFO. Regarding contrast agent preparation, 8 ml 0.9% sodium chloride solution, l ml venous blood of subjects, and 1 ml air were all put into one tube and were completely blended. In addition, at least 20 times of vibrating were required by a t-branch pipe. The participant was asked to do the Valsalva activity, in other words, to keep his breath after taking a deep breath; then, microbubbles were injected from the median cubital vein and immediately to breathe out quickly. And then whether RLS occurred, or the source of the RLS was recording. Each bubble study was performed 3 times, and the maximum number of microbubbles that was detected was used. In terms of indicators and result determination, whether microbubbles could be observed in the left heart chamber was the criterion to evaluate RLS. As for PFO-PLS, a cluster of microbubbles entered into the left heart chamber from PFO within 3 cardiac cycles after Valsalva action, while P-RLS performed that some scattered microbubbles persistently entered into the left heart chamber from the left upper pulmonary vein more than 5 cardiac cycles after Valsalva action (Figure 1).

**Figure 2:**
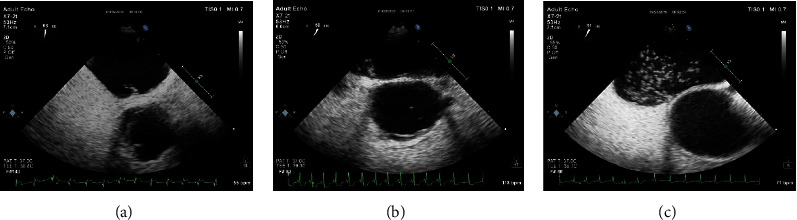
Semiquantitative grading of a patent foramen ovale-right-to-left shunt by c-TEE. (a) A small amount of RLS (1-10 microbubbles in the left atrium). (b) A moderate amount of RLS (11-30 microbubbles in the left atrium). (c) A large amount of RLS (more than 30 microbubbles in the left atrium or left atrial opacity).

Particularly, The semiquantitative grading of RLS was classified as a small amount of RLS which was 1 level, indicating 1-10 microbubbles per frame could be seen in the left atrium, a moderate amount of RLS was 2 levels, indicating 11-30 microbubbles per frame could be seen in the left atrium, and a large amount of RLS was 3 levels, indicating more than 30 microbubbles per frame, or the left atrium is filled with microvesicles [8]. The whole dynamic process of the c-TEE operation was recorded as video and saved in the diagnostic apparatus. And then two independent sonographers were required to analyze the stored video and make the corresponding diagnosis (Figure 2).

### 2.3. Statistical Analysis

A chi-square test was used to compare the ratio between the two groups. Semiquantitative shunt grading between the two methods was compared using the Wilcoxon-Mann–Whitney test. A *P* value of <0.05 indicated statistical significance. All data were analyzed using SPSS software (version 22.0, SPSS).

## 3. Results

### 3.1. Baseline Characteristics and Positive Detection Rate of Two Groups

A total of 660 patients were identified in this study. The age and gender of patients between the migraine group and the CS group were statistically different. The patients in the migraine group were younger than the CS group, and most patients in the migraine group were female. Baseline characteristics of the included patients were summarized in Table 1.

PFO-RLS was detected in 348 (348/660, 52.7%) cases by TEE, while in 392 (392/660, 59.3%) cases by c-TEE. There was a significant difference in the detection rates of PFO-RLS with TEE and c-TEE (*χ*2 = 5.95, *P* = 0.01). Simultaneously, P-RLS was detected in 239 (239/660, 36.2%) cases by c-TEE.

Among 330 patients with cryptogenic stroke, PFO-RLS was detected in 198 cases; according to the c-TTE method (198/330, 60.0%), P-RLS was detected in 111 cases (111/330, 33.6%); concurrently, 83 participants suffered from PFO-RLS and P-RLS (83/330, 25.1%), including 1 case with PFO and pulmonary arteriovenous fistula. Among 330 patients with migraine, PFO-RLS was detected in 194 cases; according to the c-TTE method (194/330, 58.7%), P-RLS was detected in 128 cases (128/330, 38.7%); simultaneously, 90 participants suffered from PFO-RLS and P-RLS (90/330, 27.2%). There were 28 cases only with P-RLS and without PFO-RLS in the cryptogenic stroke group, while 38 cases in the migraine group, which were excluded from pulmonary arteriovenous fistula by the CTA examination. There was no statistical significance of RLS detection rates between the two groups (*P* > 0.05). The RLS detection rates of the c-TEE examination in different groups were illustrated in Table 2.

### 3.2. Semiquantitative Shunt Grading

Grade 1 RLS in the migraine group (144/322) was significantly higher than that in the cryptogenic stroke group (71/309) (*P* < 0.05). Grade 3 RLS in the cryptogenic stroke group (113/309) was significantly higher than that in the migraine group (67/322) (*P* < 0.05). For grade 2 RLS, there was no statistical difference between the two groups (*P* = 0.12). Semiquantitative results on RLS originating from various sources in two groups were presented in Table 3.

## 4. Discussion

TEE is considered to be a gold indicator for diagnosing PFO, but some PFOs may have false negatives due to too long oval valve, too small foramen, the operator's improper adjustment of instruments for blood flow ruler, or two-dimensional image gain, while C-TEE can make up for these shortcomings, increase the positive rate of PFO diagnosis [9, 10], and whether the bubbles originate from the foramen ovale or the pulmonary vein are permitted to be intuitively checked. Therefore, we applied c-TEE in our study to examine the characteristics of PFO-RLS and/or P-RLS in patients with cryptogenic stroke and with migraine, and the results revealed that the positive rate of c-TEE for the right-to-left shunt of PFO compared with TEE color was significantly increased (*P* < 0.05), and the right-to-left shunt originating from the pulmonary veins can be indicated.

In the past time, cryptogenic stroke and migraine aura were the most common PFO-related neurological disorders in clinical practice. However, previous studies have shown that more than 40% of stroke patients who ever received PFO occlusion still suffered from RLS [11]. And those RLS shunt could not be imputed to the possible residual shunt of PFO and indicated the existence of P-RLS. Studies have shown that the diameter of pulmonary capillaries is 5-10 *μ*m, and there are 25-50 *μ*m arteriovenous channels in the healthy human lungs [11, 12]. However, these physiological channels are usually not open or rarely open. Only under certain physiological or pathological conditions, such as exercise, hypoxia, and even changes in body position, which will be opened and lead to P-RLS [13–16]. The previous studies demonstrated that the detection rate of P-RLS in the healthy population is 20-30% [12, 14, 17]. In our study, we followed the methods of Feng et al. 2018 [18], and our results presented that the detection rate of P-RLS was 33.6% in the CS group and 38.7% in the migraine group.

Stroke has brought serious consequences and economic burdens for families. Migraine is a common neurological disorder in clinical practice, which could seriously affect the life quality of patients. So far, contradictory microemboli can cause stroke bypass PFO, and the arteriovenous access with more than 25-50 *μ*m diameter exists in abnormal status [17, 19, 20]. Migraine is caused by contradictory microemboli and/or the nontangible components of chemical substances that can enter into systemic circulation through the open pulmonary arteriovenous physiological channels [21–23].

The quantification of RLS is another focus in many studies [17, 24]. In general, RLS identification is based on a visual evaluation of the number of contrast bubbles appearing in the left atrium at rest or during the Valsalva maneuver [25]. In our study, the semiquantitative results showed that the grades 2-3 RLS were mainly detected in the cryptogenic stroke group, while grades 1-2 RLS were mainly detected in the migraine group. In fact, individuals with migraine are at higher risk for stroke [26]. Based on our results, migraine patients with grade 2 or grade 3 RLS may be more likely to develop stroke. Therefore, active treatment such as PFO closure may prevent some stroke in subjects with PFO.

The main limitation of the current study is that poor intolerance among patients for the TEE probe and failure to perform a standard Valsalva maneuver while the TEE was being performed might affect the grading results to some extent. Therefore, during the examination, we need to train the patient's Valsalva maneuver and record three times to select the maximum number of microbubbles.

In conclusion, (1) c-TEE can increase the positive rate of PFO diagnosis compared with TEE. (2) There is no significant difference in the incidence of PFO-PLS and P-RLS between the cryptogenic stroke group and the migraine group. (3) The grades 2-3 RLS are mainly detected in the cryptogenic stroke group, while grades 1-2 RLS are mainly observed in the migraine group. Whether the patients with migraine who are detected in grades 2-3 RLS may have a stroke in the long-term requires a long-term follow-up observation.

## Figures and Tables

**Table 1:** Patient characteristics (*N* = 660).

Characteristics	Migraine group (*N* = 330)	CS group (*N* = 330)
Age (y)	39.3 ± 6.5	49.2 ± 8.6
Sex (female/male)	188/142	149/181
Diabetes (*N*, %)	14(4.2%)	21 (6.3)
Hyperlipidemia (*N*, %)	18 (5.4%)	22 (6.6%)
Hypertension (*N*, %)	13 (3.9%)	16 (4.8%)
Arrhythmia (*N*, %)	12 (3.6%)	13 (3.9%)

Age and sex were statistically significant in the two groups (*P* < 0.05); other characteristics were not statistically significant in the two groups.

**Table 2:** Comparisons of RLS detection rates of c-TEE examination in two groups.

	CS group (*N* = 330)	Migraine group (*N* = 330)
Only PFO-RLS (*N*, %)	115 (34.8)	104 (31.5)
PFO-RLS + P-RLS (*N*, %)	83(25.1)	90 (27.2)
Only P-RLS (*N*, %)	28 (8.4)	38 (11.5)

**Table 3:** Semiquantitative grading of RLS originating from various sources in two groups.

Group	Source	Grade 1	Grade 2	Grade 3
CS group	PFO-RLS (198)	45	80	73
	P-RLS (111)	26	45	40

Migraine group	PFO-RLS (194)	89	71	34
	P-RLS (128)	55	40	33

## Data Availability

The materials in this manuscript are available from the corresponding author on reasonable request.
